# Deflection, Frequency, and Stress Characteristics of Rectangular, Triangular, and Step Profile Microcantilevers for Biosensors

**DOI:** 10.3390/s90806046

**Published:** 2009-07-29

**Authors:** Mohd Zahid Ansari, Chongdu Cho

**Affiliations:** Department of Mechanical Engineering, Inha University, 253 Yonghyun-dong, Nam-Ku, Incheon, 402-751, Korea

**Keywords:** biosensor, surface stress, microcantilever, resonant frequency, deflection

## Abstract

This study presents the deflection, resonant frequency and stress results of rectangular, triangular, and step profile microcantilevers subject to surface stress. These cantilevers can be used as the sensing element in microcantilever biosensors. To increase the overall sensitivity of microcantilever biosensors, both the deflection and the resonant frequency of the cantilever should be increased. The effect of the cantilever profile change and the cantilever cross-section shape change is first investigated separately and then together. A finite element code ANSYS Multiphysics is used and solid finite elements cantilever models are solved. A surface stress of 0.05 N/m was applied to the top surface of the cantilevers. The cantilevers are made of silicon with elastic modulus 130 GPa and Poisson’s ratio 0.28. To show the conformity of this study, the numerical results are compared against their analytical ones. Results show that triangular and step cantilevers have better deflection and frequency characteristics than rectangular ones.

## 1. Introduction

The ability of label-free detection, scalability to allow massive parallelization, and sensitivity of the detection range applicable to *in vivo* problems are some of the important requirements for a future generation of biosensors [1]. Although generally used in the topological investigations of surfaces such as in atomic force microscopy, arrays of microcantilevers are attracting much interest as sensors in a variety of applications. Microcantilever sensors have emerged as a universal, very powerful and highly sensitive tool to study various physical, chemical, and biological phenomena. As biosensors, they are found to display label-free, real-time and rapid assaying features [1–7]. Microcantilever biosensors generally use optical deflection readout technique to measure the adsorbate-induced deflections, and hence in assaying the unknown species present in a media. In microcantilever biosensors, the accuracy of measurement strongly depends on the accurate determination of the surface stress induced deflections. The deflections usually range a few tens to a few hundreds of a nanometre. Measuring deflections of this order requires extremely sophisticated readout arrangements. Therefore, increasing the sensitivity of a microcantilever without increasing the complexity in the deflection detection is a major challenge.

The overall sensitivity of a microcantilever biosensor depends on the design sensitivity of the cantilever and the measurement sensitivity of the deflection measurement system. A sensitive cantilever design should efficiently convert the biomolecular stimulus into a large cantilever deflection, whereas the measurement sensitivity should ensure that the deflections measured are only induced because of the biomolecular stimulus and not due to some ambient disturbance source. The design sensitivity of the cantilever can be improved by changing the cantilever design in such a way that for a given surface stress larger deflections can occur. This scheme can be realized by reducing the bending stiffness of the cantilever [8–11] or by using softer cantilever materials [7,12–15]. To improve the measurement sensitivity, the fundamental resonant frequency of the cantilevers should be made as large as possible, because the accuracy in deflection measurement depends not only on the deflection occurred, but also on the signal-to-noise ratio. Most of the noise in the deflection signal can be attributed to flow [16] and thermal [17,18] induced excitations. To improve the signal-to-noise ratio, and hence the measurement sensitivity, the resonant frequency of the cantilever should be made as high as possible. Thus, to increase the overall cantilever sensitivity, we should select a design that exhibits both higher deflection and higher resonant frequency.

To improve the design sensitivity of cantilevers various designs and schemes have been reported. Silicon microcantilevers are commonly used in biosensors. However, due to high elastic modulus silicon cantilevers exhibit extremely low deflections for a given surface stress change. Therefore, to increase the deflections polymer cantilevers can be used. Since the elastic modulus of polymers is generally much lower than silicon, the deflections induced are magnified many folds. Polymer cantilevers, however, have a major drawback in being very temperature sensitive, because of the thermal bimetallic effects. Thermal induced deflections exceeding the surface-stress induced deflections are not uncommon. Hence, polymer cantilevers require a fine control of the surrounding. The other way to improve design sensitivity is to increase the cantilever deflection by changing the shape of the cantilever. By reducing the moment of inertia of a cantilever its bending stiffness can be reduced, which results in higher deflection.

With the objective of increasing the deflection and resonant frequency at the same time, this paper investigates the deflection and vibration analysis of rectangular, triangular, and step profile microcantilevers having basic and modified shapes. The surface-stress induced deflection in the microcantilever is modelled by an equivalent in-plane tensile force acting on the top surface of the cantilever, in the length direction. A commercial finite element method (FEM) software ANSYS Multiphysics is used in this analysis. All the cantilevers are investigated for deflection, fundamental resonant frequency and stress.

## 2. Theory

The surface stresses, in general, are generated either by the redistribution of the electronic charge at the surface, due to the change in the equilibrium positions of the atoms near the surface, or by the adsorbtion of foreign atoms onto its surface to saturate the dangling bonds [19]. Microcantilever biosensors exploit the surface-stress induced deflections to assay the target molecules. When the target molecules attach onto the functionalized top surface of the cantilever, the surface stress distribution on this surface is changed, resulting in a differential stress across the top and bottom surfaces of the cantilever. The differential stress ultimately generates deflections in the cantilever.

**Figure 1.**
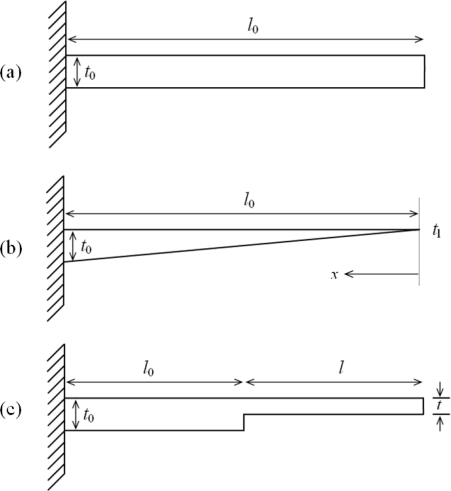
Schematic designs of (a) rectangular, (b) triangular, and (c) step profile cantilevers.

For a rectangular profile microcantilever (Figure 1a), the differential surface stress (Δ*σ*) and deflection (Δ*z*) are related by the Stony Equation [20] given as:

(1) $$\Delta z=\;\;\frac{4(1-\nu )\;\Delta \sigma }{E}{\left(\frac{l_{0}}{t_{0}}\right)}^{2}$$

where *l*_0_ and *t*_0_ are the length and thickness of the cantilever, and *E* and *ν* are the elastic modulus and Poisson’s ratio of the cantilever material. The fundamental resonant frequency (*f*_0_) for a rectangular profile cantilever of mass density (*ρ*) [21] can be is given as:

(2) $$f_{0}=\;\;\frac{1}{2\pi }\sqrt{\frac{E}{\rho }}\cdot \frac{t_{0}}{l_{0}^{2}}$$

As can be seen from Equations 1 and 2, any attempt to increase the deflection by increasing the length or decreasing the thickness will decrease the resonant frequency. In fact, the two equations indicate an inverse relationship between them. For instance, following Equation 1, if we try to increase the deflection by increasing the length or decreasing the thickness, Equation 2 predicts an opposite effect for the frequency. Thus, the deflection and frequency are coupled terms, and hence, should be treated in such manner. Combining Equations 1 and 2, we define overall sensitivity (Δ*z·f*_0_) term as:

(3) $$\Delta z\cdot f_{0}=\;\;\frac{2(1-\nu )\;\Delta \sigma }{\pi \sqrt{E\rho }}\cdot \frac{1}{t_{0}}$$

It should be, however, noticed in Equation 3 that cantilever thickness cannot be changed arbitrarily, because achieving an economical and viable microfabrication process and assuring the structural and functional reliability of the cantilever puts a limit on selecting the minimum thickness. Equation 3 suggests that instead of increasing deflection or resonant frequency individually, it is more practical to increase the overall sensitivity. Therefore, comparing Δ*z·f*_0_ values is a better way to compare the performance of a particular microcantilever design, because depending on this value appropriate cantilever dimensions and characteristics can be selected. To select the best cantilever model, we should choose one that has higher Δ*z·f*_0_ value, more inclined towards increased deflection. Therefore, in this study we calculated and compared the sensitivity values of all the cantilever models. For achieving higher deflection, we should choose longer cantilevers (Equation 1), whereas, for higher frequency we should choose shorter ones (Equation 2).

Since it is not possible to achieve zero tip thickness for a cantilever, the triangular profile can be approximated as a trapezoidal profile, of the form *t*(*x*) = *t*_l_ + (*t*_0_ – *t*_l_) *x*/*l*, having very small tip thickness (Figure 1b).

The Stoney Equation for a triangular profile cantilever can be given as [10]:

(4) $$\Delta z=\;\;\frac{8\;(1-\nu )\;\Delta \sigma l^{2}}{E{\left(t_{0}-t_{l}\right)}^{2}}\left[\text{ln}\left(\frac{t_{0}}{t_{l}}\right)+\frac{t_{l}}{t_{0}}-1\right]$$

where *t*_0_ and *t*_l_ are the thicknesses of the cantilever at the fixed and free ends. Hoffman and Wertheimer [22] gave a simple and accurate formula for calculating the fundamental resonant frequency for a beam of triangular profile:

(5) $$f_{0}=\;\;C\sqrt{\frac{S}{M}}$$

where *S* and *M* are spring constant and mass of the cantilever; and *C* is taper-ratio dependent mass distribution parameter.

By using Euler beam theory and principle of superposition for nonprismatic beams [23], the Stoney equation for a step profile cantilever (Figure 1c) can be modified to give:

(6) $$\Delta z=\;\;\frac{4\;(1-\nu )\;\Delta \sigma }{E}{\left[{\left(\frac{l_{0}}{t_{0}}\right)}^{3}+\frac{\left(3l_{0}+2l\right)l^{2}}{2t^{3}}\right]}^{2/3}$$

where *l*_0_ and *l* are lengths of the thick and thin sections of the cantilever. And its fundamental resonant frequency can be given as:

(7) $$f_{0}=\;\;\frac{1}{2\pi }\sqrt{\frac{{\mathrm{Et}}_{0}^{3}}{\rho \left(l_{0}t_{0}+lt\right){\left(l_{0}+l\right)}^{3}}}$$

## 3. Modelling and Simulation

The surface-stress induced deflection in a microcantilever can be modelled by applying a lengthwise in-plane tensile force at the free end of the top surface of the cantilever [8]. The simulations assumed the cantilevers are made of silicon, and have an elastic modulus of 130 GPa and a Poisson’s ratio of 0.28, respectively. The cantilevers are subject to a surface stress (Δ*σ*) of 0.05 N/m on their top surfaces. Since surface stress is expressed in unit of force per unit width, multiplying the surface stress by the cantilever width will give the total tensile force acting on the top surface. And, therefore a tensile force of *F* = 0.05 N/m × 100 × 10^−6^ m = 5 × 10^−6^ N was applied to the top free edge of all the six models.

**Figure 2.**
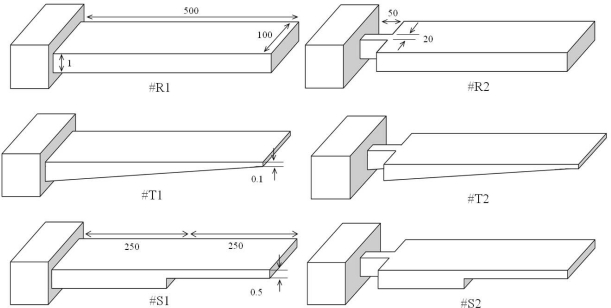
Comparison between basic and modified rectangular (R), triangular (T), and step (S) profile cantilevers. All the models have equal length, width, and fixed-end thickness (unit: micrometre).

Figure 2 shows a comparison between basic and modified designs for rectangular, triangular, and step profile cantilevers analyzed in this study. As can be seen in the figure, the basic designs have uniform width throughout their entire length (i.e., #R1, #T1, and #S1). The modified designs have their widths reduced towards the fixed end (i.e., #R2, #T2, and #S2), and are connected to the fixed end by a 50 μm long and 20 μm wide strip. All the cantilevers are 500 μm long, 100 μm wide, and 1 μm thick at their fixed-ends. In step profile cantilevers, the thin section thickness is half the thick section, and both the sections have equal length, i.e. *t* = *t*_0_/2 and *l* = *l*_0_. In triangular cantilevers, the free end thickness is one-tenth the fixed end. Simulations used FEM software ANSYS Multiphysics to calculate the deflection, fundamental resonant frequency and stress induced in all the six designs. The simulations were performed on three-dimensional FE models of the cantilevers, under linear and static conditions. In simulations, we used micrometre as unit of length and newton of force (Figure 4). Mesh size convergence test was performed to eliminate any mesh size effect on the analysis. The FE models were meshed by SOLSH190 elements.

## 4. Results and Discussion

**Figure 3.**
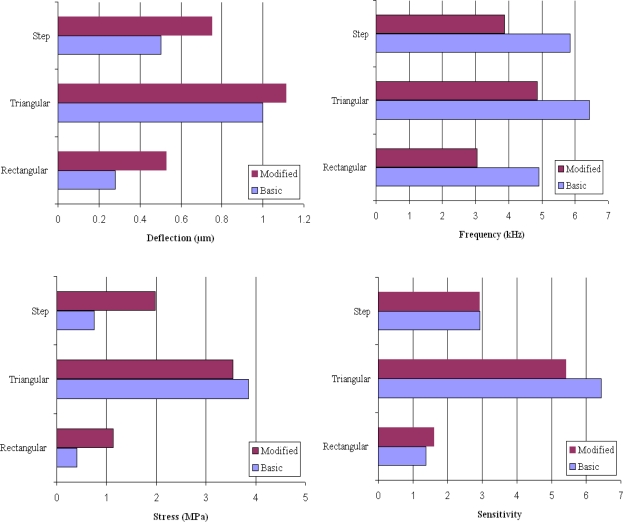
Results showing deflection, frequency, stress, and sensitivity values for the basic and modified design rectangular, triangular, and step profile cantilevers.

Table 1 shows a comparison between the analytical and simulation results for maximum deflection and resonant frequency of basic rectangular, triangular, and step profile cantilevers. For calculating the deflection and frequency values for the rectangular cantilever (#R1), Equations 1 and 2 were used. For triangular cantilever Equations 4 and 5, and for step cantilever Equations 6 and 7 were used. As can be observed in Table 1, the analytical and simulation values for all cantilever types show comparable results, indicating the conformity of the simulation analysis. Figure 3 shows the simulation results for the microcantilever designs analyzed in this study.

Table 2 lists the normalized simulation results for the maximum deflection (Δ*z*), fundamental resonant frequency (*f*_0_), overall sensitivity (Δ*z · f*_0_), and maximum stress induced (*σ*_max_) for all the six models shown in Figure 2. The results for #R1 are used to normalize the results for the remaining models. The four values for #R1 are 0.28 μm, 4.91 kHz, 1.37, and 0.41 MPa, respectively. From Table 2 it is obvious that the design sensitivity of cantilevers can be improved by simply changing the cantilever profile. For instance, comparing the deflections indicated by #R1 with #T1 and #S1, we easily observe that deflections are increased by 257% and 79%, respectively. Thus, we can improve design sensitivity of the cantilevers used in biosensor by replacing the rectangular profile cantilever by a triangular or step profile cantilever. In can be further noticed in Table 2 that by changing the basic shapes of the rectangular, triangular, and step profile cantilevers, the induced deflections can be further improves. For instance, by changing the design from #R1 to #R2, #T1 to #T2, and #S1 to #S2 the deflections induced are improved by about 89%, 11%, and 50%, respectively. However, if we combine both the profile and the shape change of the cantilever designs, we observe the deflections are improved by 296% for #T2 and 168% for #S2 designs than the conventional design #R1. Since higher deflections indicate higher design sensitivity of the cantilever design, we may conclude that the design sensitivity of the microcantilever used in biosensors can by improved changing the cantilever profile and/or shape to triangular or step designs.

The deflections are observed to increase in all the cases whether the profile is changed, cross-section shape is changed, or both are changed (Figure 3). This behaviour is not unexpected and can be explained by the role of bending stiffness of the cantilevers, because in all the cases the bending stiffness is reduced. In general, the bending stiffness of a cantilever depends on its cross-sectional area at the fixed end. The higher the cross-sectional area, the higher the stiffness will be. But, it should be noted that for a given cross-sectional area at the fixed end, thicker cantilevers will have higher stiffness than the thinner one. In other words, the bending stiffness of a cantilever can be reduced by reducing the cantilever thickness or the cantilever cross-sectional area at the fixed end. In our study the fixed end thickness is kept constant at 1 μm, and the stiffness is reduced by either changing the profile or reducing the cross-sectional of the cantilever designs (Figure 2). The effect of profile and cross-section shape change on deflection is discussed next.

The major difference between #R1, #T1, and #S1 designs is that in #R1 the bending stiffness is constant along the cantilever length, whereas in both #T1 and #S1 it is not. When we changed the cantilever profile, we basically changed the thickness of the cantilever towards the free end (i.e., #T1 and #S1), which reduced the bending stiffness. It should be noticed that in #T1 the thickness is reduced continuously along the cantilever length, and therefore, the bending stiffness is reduced continuously along the length. In case of step profile (#S1), since both the thick and thin sections have constant thickness, the stiffness is constant in both sections. Since the thickness of thin section is half the thick section, its stiffness is lower and therefore larger deflection will occur in thin section. Among the three basic profiles, since #T1 has the least thickness at free end, it shows the maximum tip deflection, see Table 2. In case of #R2, #T2, and #S2 the cross-sectional area at the fixed end are changed. And as expected, the reduction is area further augmented the deflections.

As discussed before, the dynamic properties of microcantilevers used in biosensors are critical in accurate measurement of deflections. In practical applications, there can be thermal, structural, or flow induced excitations that can interfere with and hence produce noise in the signals. Therefore, it is vital to eliminate or isolate the noise in the signal, and to insure that the deflections induced are solely due to the surface stress change. To prevent noise, a cantilever should have high signal-to-noise ratio, which can be achieved by making the resonant frequency of the cantilever as high as possible. The higher the resonant frequency, the higher the measurement sensitivity will be. In Table 2, we observe that changing the cantilever profile from #R1 to #T1 and #S1 improved the resonant frequency of the cantilevers by 31% and 19%, respectively. In other words, by changing the cantilever profile, the measurement sensitivity is improved. However, when we try to improve the frequency by changing the design from #R1 to #R2, #T1 to #T2, and #S1 to #S2, we observe that in all the cases the frequencies are reduced by about 38%, 24%, and 37%, respectively. This behaviour suggests that the shape change has adverse effects on the frequency characteristics of the cantilevers.

The improved resonant frequencies due to profile change observed in Table 2 can be explained by the reduction in mass of the cantilevers. The resonant frequency of a cantilever is directly proportional to the square root of bending stiffness and inversely proportional to the square root of cantilever mass. In other words, the frequency can be improved by increasing the cantilever stiffness and/or reducing the cantilever mass. It is obvious in Figure 2 that #T1 and #S1 have respectively 45% and 25% less mass than #R1, and therefore show respectively 31% and 19% higher frequency than #R1. The frequency results for #R2, #T2, and #S2 depict a different picture. The reduction in fixed-end area of the cantilevers reduced the frequency. This observation is not unexpected. Since the frequency is proportional to square root of stiffness, by reducing the cross-sectional area at the fixed end basically we reduced the stiffness of the cantilever, which resulted in reduced frequencies. Thus we see that the area reduction, on one hand, increased the deflections, but on the other, reduced the frequencies. Higher deflection and higher resonant frequency are critical in optimal performance of microcantilever biosensors. Deflections affect the design sensitivity and frequencies affect the measurement sensitivity of the microcantilever biosensors. Therefore, for comparing the performance of different cantilever schemes, we defined the term overall sensitivity expressed by Δ*z*·*f*_0_ value.

In Table 2, by changing the profile the overall sensitivity (Δ*z*·*f*_0_) values of the basic cantilever designs are improved by 368% for #T1 and 113% for #S1 from conventional design #R1. However, the reduction in cross-sectional area has mixed effect on the sensitivity values. For instance, the area change improved the sensitivity by 17% for #R2, but reduced by 15% for #T2. In case of #S2 the area change has almost no effect on the overall sensitivity. Since in case of #T2 the sensitivity is reduced by 15%, mainly because of the reduction in frequency, we can conclude that #T2 has no significant advantage over #T1. Therefore, the basic triangular design should be preferred. If we compare the rectangular and step designs, we observe that #S2 has big advantage over #R1 and #R2 in terms of both greater deflection and higher sensitivity. And therefore, #S2 design should be preferred.

**Figure 4.**
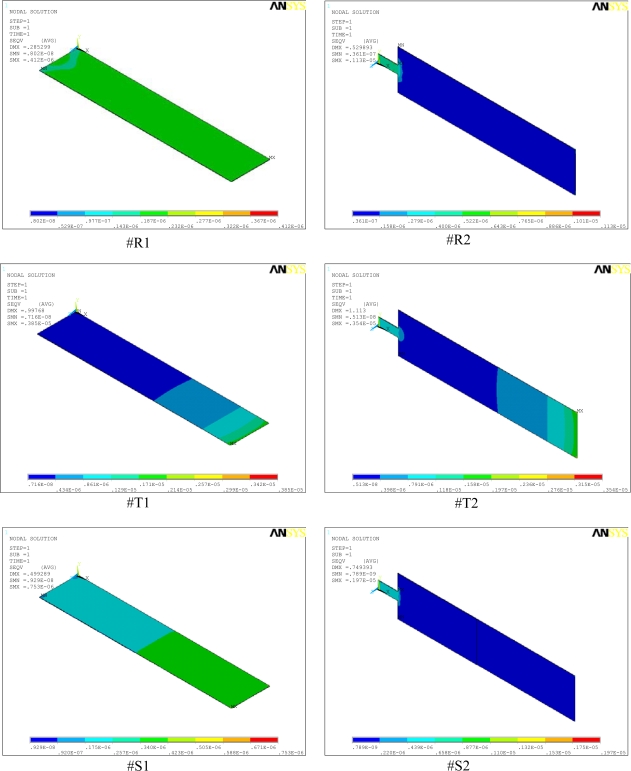
Stress distributions in the rectangular (#R1, #R2), triangular (#T1, #T2), and step (#S1, #S2) profile microcantilevers.

Figure 4 shows the simulation results for stress distribution in all the six designs analyzed in this study. The maximum stress (SMX) and the maximum deflections (DMX) values are indicated in the top-left corner of the micrographs. In the analysis, we used micrometre as the unit of length and newton of force. Accordingly, in the figure, the cantilever size and deflections are expressed in micrometres and the stresses in TPa (i.e., 10^6^ MPa). A comparison between the stress values of #R1, #T1, and #S1 shows that profile change alone increased the stresses from 0.41 to 3.85 and 0.75 MPa, respectively (Figure 4). The maximum induced stresses ranges from a minimum of 0.41 MPa for #R1 to a maximum of 3.85 MPa for #T1. Stress analysis results indicate that since the induced stresses are much less than the ultimate strength of 300 MPa for silicon [24], we may conclude that all the cantilevers designs are safe.

Thus, we see that both #T1 and #S2 show both greater deflection and higher sensitivity than the rest designs, and therefore should be the preferred microcantilever designs for improving the biosensor performance. However, in terms of practical applications, #S2 has big advantage over #T1, because the latter is very difficult to fabricate. For instance, microfabrication of a triangular profile cantilever of taper 1 μm to 0.1 μm is extremely difficult. In addition, ensuring the structural and functional reliability of such a thin cantilever is very challenging. On the other hand, the dimensions of #S2 analyzed in this study can be easily fabricated in silicon using deep reactive ion etching (DRIE). Therefore, we may conclude that though the performance of #T1 seems much improved than #S2, for practical application #S2 is better.

## 5. Conclusions

Arrays of microcantilevers are increasingly being used as physical, biological, and chemical sensors in various applications. In this work, we investigated improving the overall sensitivity of the microcantilevers that can be used in biosensors by increasing their deflection and frequency characteristics of the cantilevers. To improve the sensitivity we studied basic and modified design rectangular, triangular, and step profile microcantilevers. The overall sensitivity of microcantilever depends on both the deflection and the resonant frequency of the cantilever. The simulation results obtained in this study correspond well to their analytical models, validating the conformity of the analyses. The surface stress was successfully modelled by an in-plane tensile force applied to the top surface of the cantilevers. We found that by changing the profile from rectangular to triangular and step, the cantilever deflections are improved by 257% and 79%, and frequencies by 31% and 19%, respectively. Further, for each cantilever type, the cross-section shape change by reducing fixed-end area increased the deflection by 89%, 11%, and 50%, but reduced the frequencies 38%, 24%, and 37%, respectively. The overall sensitivity values of all the cantilevers are improved, however. Though the triangular profile cantilevers showed better deflection and frequency characteristics, fabrication and structural integrity constraints suggest that a step cantilever (#S2) is more practical as the sensing element of the biosensor. We also found that compared to the excellent mechanical properties of silicon, the maximum stress induced in the designs are negligible.

## Figures and Tables

**Table 1.** Comparison between analytical and simulation results for basic rectangular, triangular, and step cantilevers.

**Model**	**Max. Deflection (μm)**		**Frequency (kHz)**	

	**Analytical**	**Simulation**	**Analytical**	**Simulation**

#R1	0.28	0.28	4.79	4.91
#T1	0.96	1.00	7.30	6.44
#S1	0.53	0.50	5.53	5.84

**Table 2.** Comparison between normalized values for maximum deflection, fundamental resonant frequency, sensitivity and maximum induced stress.

**Model**	***Δz***	***f****_0_*	***Δz*·*f_0_***	***σ*_max_**

#R1	1	1	1	1
#R2	1.89	0.62	1.17	2.76
#T1	3.57	1.31	4.68	9.39
#T2	3.96	0.99	3.92	8.65
#S1	1.79	1.19	2.13	1.83
#S2	2.68	0.79	2.12	4.83
